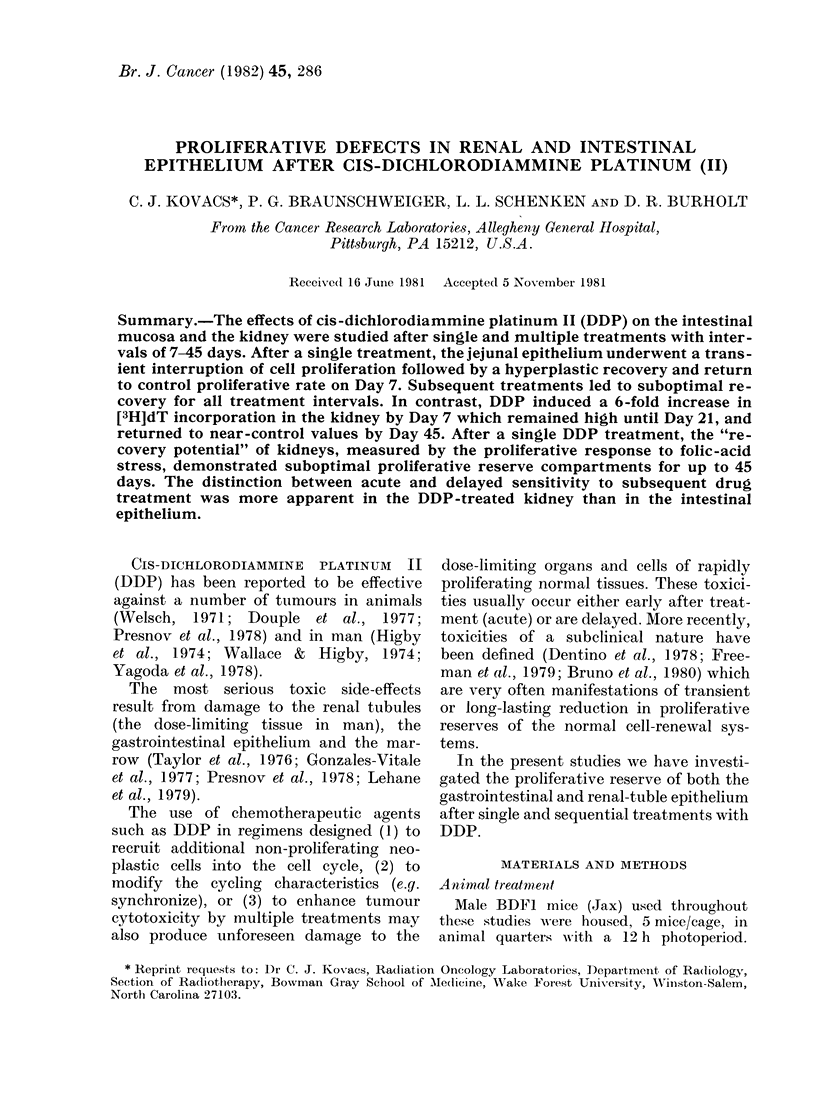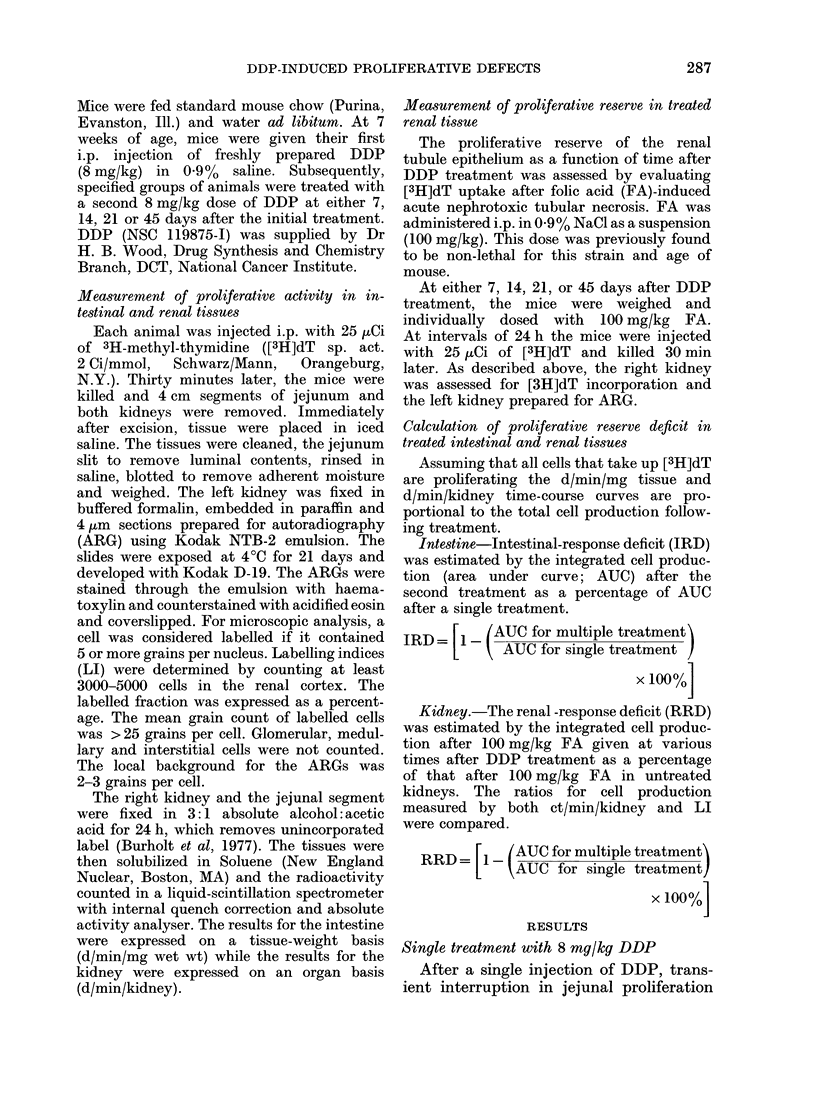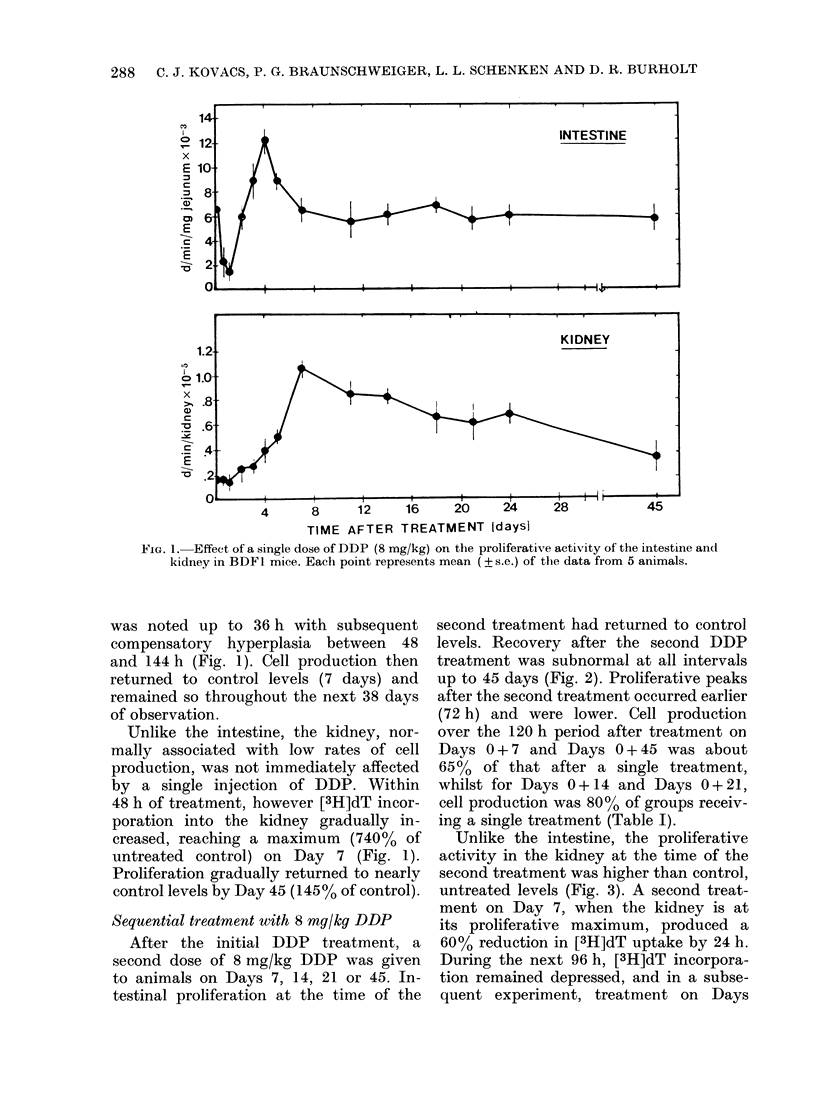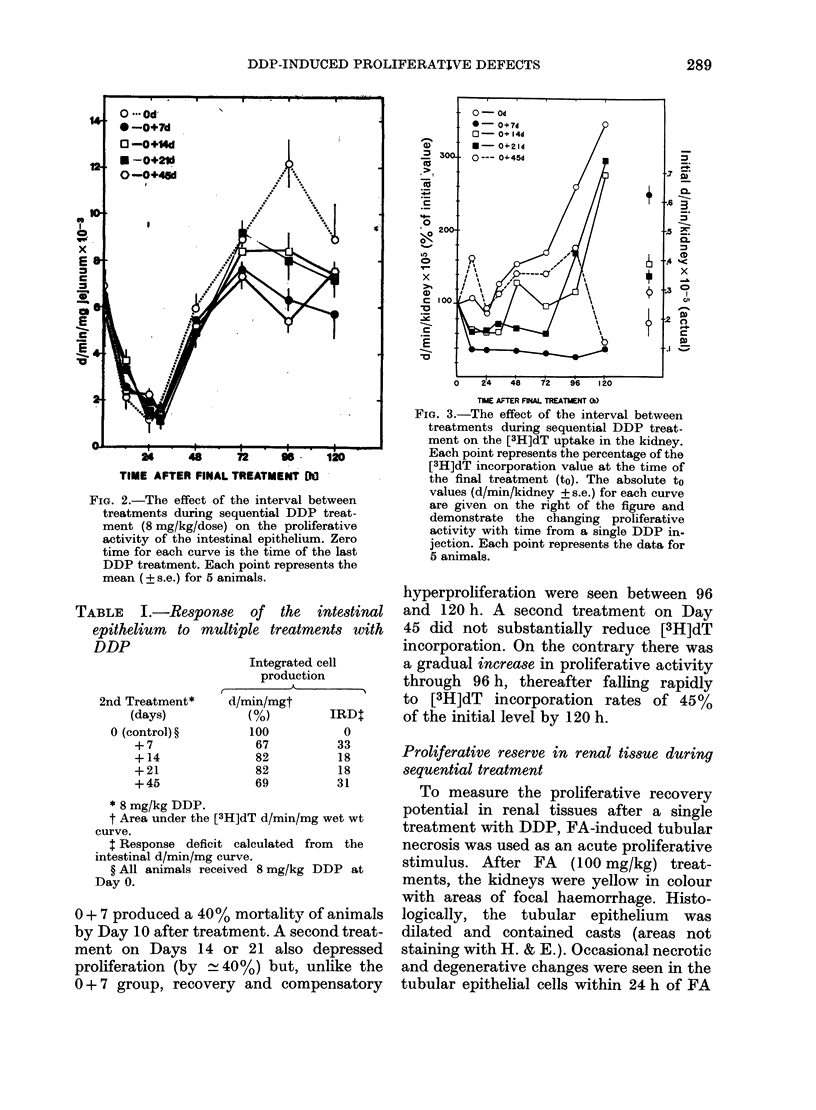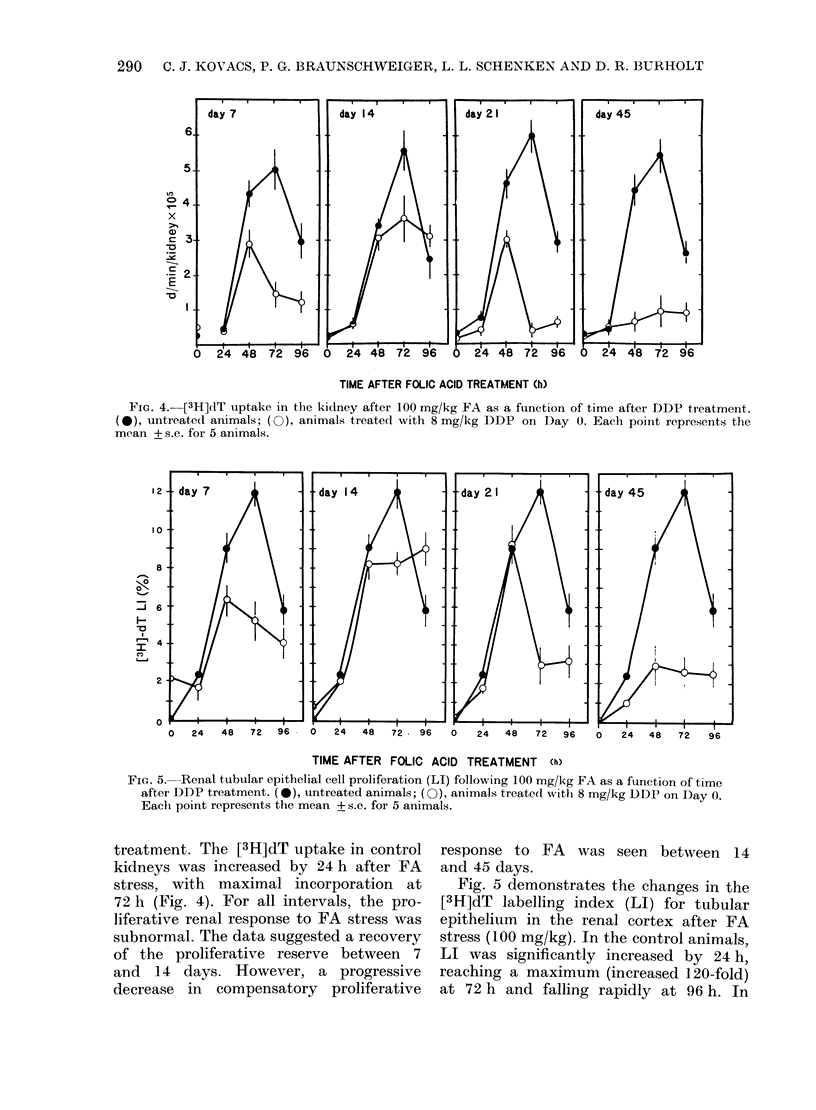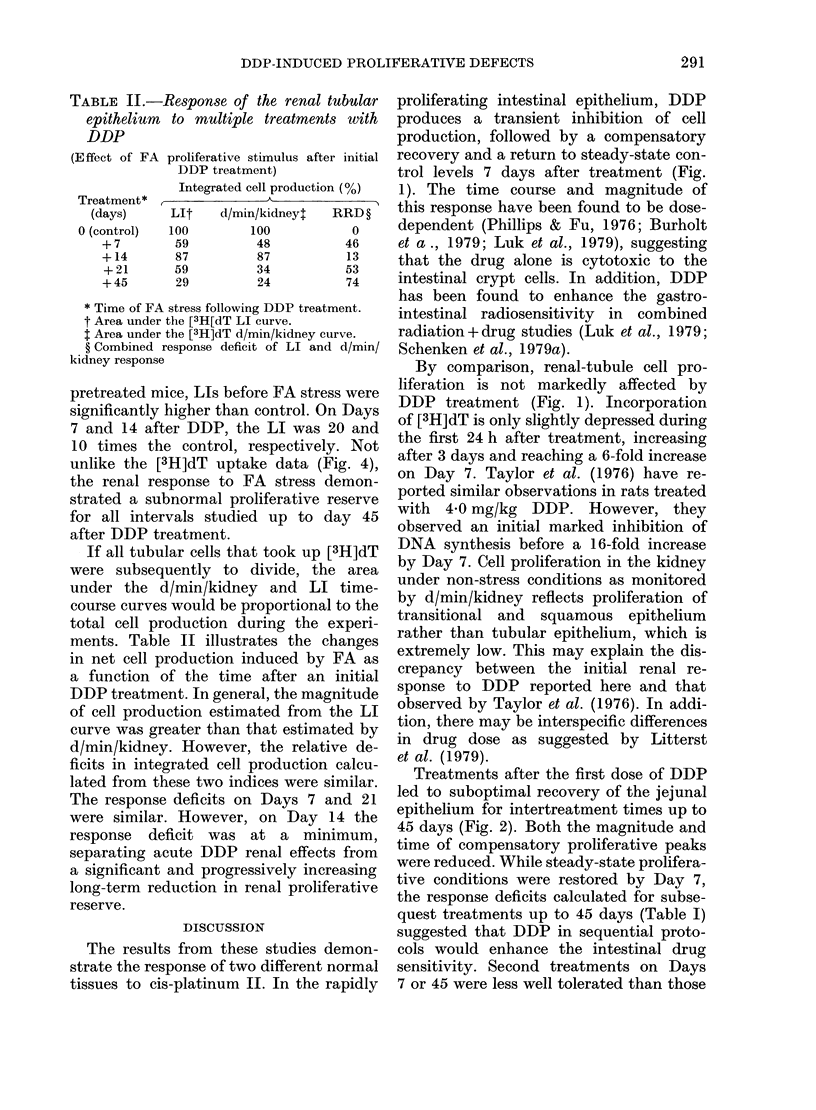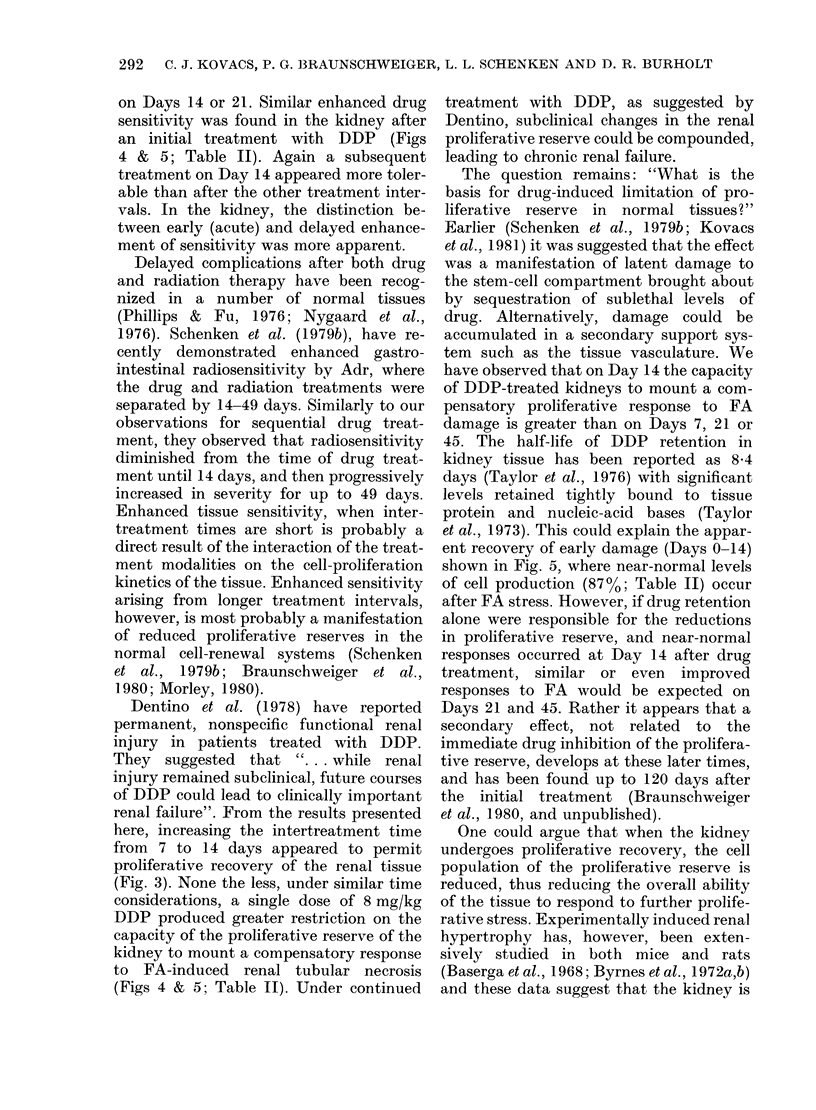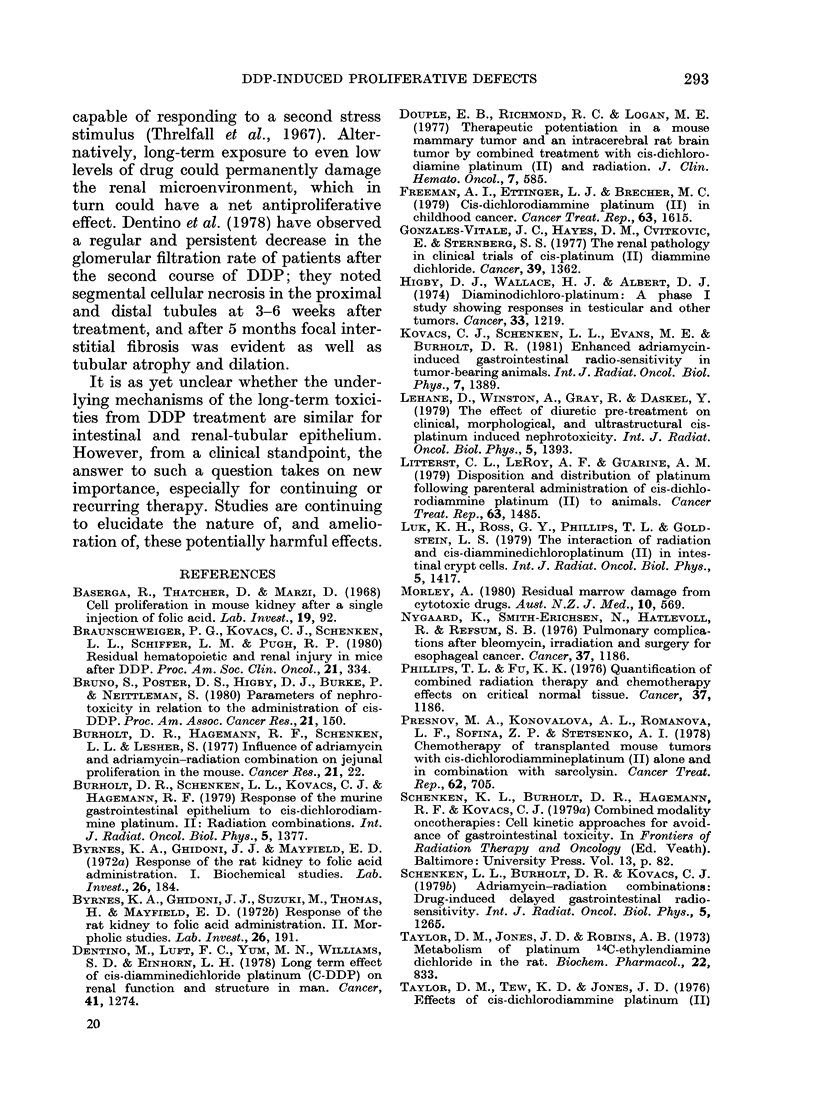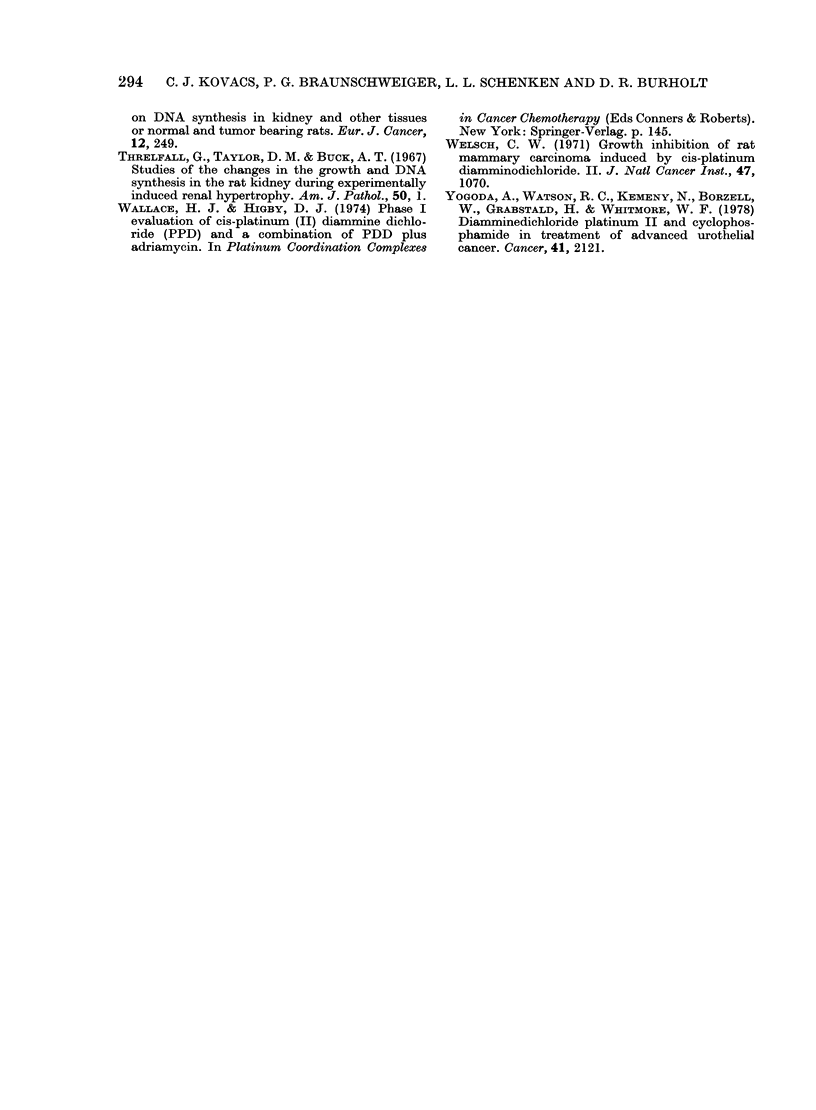# Proliferative defects in renal and intestinal epithelium after cis-dichlorodiammine platinum (II).

**DOI:** 10.1038/bjc.1982.45

**Published:** 1982-02

**Authors:** C. J. Kovacs, P. G. Braunschweiger, L. L. Schenken, D. R. Burholt

## Abstract

The effects of cis-dichlorodiammine platinum II (DDP) on the intestinal mucosa and the kidney were studied after single and multiple treatments with intervals of 7-45 days. After a single treatment, the jejunal epithelium underwent a transient interruption of cell proliferation followed by a hyperplastic recovery and return to control proliferative rate on Day 7. Subsequent treatments led to suboptimal recovery for all treatment intervals. In contrast, DDP induced a 6-fold increase in [3H]dT incorporation in the kidney by Day 7 which remained high until Day 21, and returned to near-control values by Day 45. After a single DDP treatment, the "recovery potential" of kidneys, measured by the proliferative response to folic-acid stress, demonstrated suboptimal proliferative reserve compartments for up to 45 days. The distinction between acute and delayed sensitivity to subsequent drug treatment was more apparent in the DDP-treated kidney than in the intestinal epithelium.


*Br. J. Cancer* (1982) **45**, 286

### PROLIFERATIVE DEFECTS IN RENAL AND INTESTINAL EPITHELIUM AFTER CIS-DICHLORODIAMMINE PLATINUM (II)

C. J. KOVACS*, P. G. BRAUNSCHWEIGER, L. L. SCHENKEN AND D. R. BURHOLT

*From the Cancer Research Laboratories, Allegheny General Hospital, Pittsburgh, PA* 15212, *U.S.A.*

Received 16 June 1981 Accepted 5 November 1981

**Summary.—The effects of cis-dichlorodiammine platinum II (DDP) on the intestinal mucosa and the kidney were studied after single and multiple treatments with intervals of 7–45 days. After a single treatment, the jejunal epithelium underwent a transient interruption of cell proliferation followed by a hyperplastic recovery and return to control proliferative rate on Day 7. Subsequent treatments led to suboptimal recovery for all treatment intervals. In contrast, DDP induced a 6-fold increase in [$^3$H]dT incorporation in the kidney by Day 7 which remained high until Day 21, and returned to near-control values by Day 45. After a single DDP treatment, the "recovery potential" of kidneys, measured by the proliferative response to folic-acid stress, demonstrated suboptimal proliferative reserve compartments for up to 45 days. The distinction between acute and delayed sensitivity to subsequent drug treatment was more apparent in the DDP-treated kidney than in the intestinal epithelium.**

CIS-DICHLORODIAMMINE PLATINUM II (DDP) has been reported to be effective against a number of tumours in animals (Welsch, 1971; Douple *et al.*, 1977; Presnov *et al.*, 1978) and in man (Higby *et al.*, 1974; Wallace & Higby, 1974; Yagoda *et al.*, 1978).

The most serious toxic side-effects result from damage to the renal tubules (the dose-limiting tissue in man), the gastrointestinal epithelium and the marrow (Taylor *et al.*, 1976; Gonzales-Vitale *et al.*, 1977; Presnov *et al.*, 1978; Lehane *et al.*, 1979).

The use of chemotherapeutic agents such as DDP in regimens designed (1) to recruit additional non-proliferating neoplastic cells into the cell cycle, (2) to modify the cycling characteristics (*e.g.* synchronize), or (3) to enhance tumour cytotoxicity by multiple treatments may also produce unforeseen damage to the dose-limiting organs and cells of rapidly proliferating normal tissues. These toxicities usually occur either early after treatment (acute) or are delayed. More recently, toxicities of a subclinical nature have been defined (Dentino *et al.*, 1978; Freeman *et al.*, 1979; Bruno *et al.*, 1980) which are very often manifestations of transient or long-lasting reduction in proliferative reserves of the normal cell-renewal systems.

In the present studies we have investigated the proliferative reserve of both the gastrointestinal and renal-tuble epithelium after single and sequential treatments with DDP.

### MATERIALS AND METHODS

### *Animal treatment*

Male BDF1 mice (Jax) used throughout these studies were housed, 5 mice/cage, in animal quarters with a 12 h photoperiod.

* Reprint requests to: Dr C. J. Kovacs, Radiation Oncology Laboratories, Department of Radiology, Section of Radiotherapy, Bowman Gray School of Medicine, Wake Forest University, Winston-Salem, North Carolina 27103.

Mice were fed standard mouse chow (Purina, Evanston, Ill.) and water *ad libitum*. At 7 weeks of age, mice were given their first i.p. injection of freshly prepared DDP (8 mg/kg) in 0·9% saline. Subsequently, specified groups of animals were treated with a second 8 mg/kg dose of DDP at either 7, 14, 21 or 45 days after the initial treatment. DDP (NSC 119875-I) was supplied by Dr H. B. Wood, Drug Synthesis and Chemistry Branch, DCT, National Cancer Institute.

*Measurement of proliferative activity in intestinal and renal tissues*

Each animal was injected i.p. with 25 $\mu$Ci of $^{3}$H-methyl-thymidine ([$^{3}$H]dT sp. act. 2 Ci/mmol, Schwarz/Mann, Orangeburg, N.Y.). Thirty minutes later, the mice were killed and 4 cm segments of jejunum and both kidneys were removed. Immediately after excision, tissue were placed in iced saline. The tissues were cleaned, the jejunum slit to remove luminal contents, rinsed in saline, blotted to remove adherent moisture and weighed. The left kidney was fixed in buffered formalin, embedded in paraffin and 4 $\mu$m sections prepared for autoradiography (ARG) using Kodak NTB-2 emulsion. The slides were exposed at 4°C for 21 days and developed with Kodak D-19. The ARGs were stained through the emulsion with haematoxylin and counterstained with acidified eosin and coverslipped. For microscopic analysis, a cell was considered labelled if it contained 5 or more grains per nucleus. Labelling indices (LI) were determined by counting at least 3000–5000 cells in the renal cortex. The labelled fraction was expressed as a percentage. The mean grain count of labelled cells was >25 grains per cell. Glomerular, medullary and interstitial cells were not counted. The local background for the ARGs was 2–3 grains per cell.

The right kidney and the jejunal segment were fixed in 3:1 absolute alcohol:acetic acid for 24 h, which removes unincorporated label (Burholt *et al*, 1977). The tissues were then solubilized in Soluene (New England Nuclear, Boston, MA) and the radioactivity counted in a liquid-scintillation spectrometer with internal quench correction and absolute activity analyser. The results for the intestine were expressed on a tissue-weight basis (d/min/mg wet wt) while the results for the kidney were expressed on an organ basis (d/min/kidney).

*Measurement of proliferative reserve in treated renal tissue*

The proliferative reserve of the renal tubule epithelium as a function of time after DDP treatment was assessed by evaluating [$^{3}$H]dT uptake after folic acid (FA)-induced acute nephrotoxic tubular necrosis. FA was administered i.p. in 0·9% NaCl as a suspension (100 mg/kg). This dose was previously found to be non-lethal for this strain and age of mouse.

At either 7, 14, 21, or 45 days after DDP treatment, the mice were weighed and individually dosed with 100 mg/kg FA. At intervals of 24 h the mice were injected with 25 $\mu$Ci of [$^{3}$H]dT and killed 30 min later. As described above, the right kidney was assessed for [3H]dT incorporation and the left kidney prepared for ARG.

*Calculation of proliferative reserve deficit in treated intestinal and renal tissues*

Assuming that all cells that take up [$^{3}$H]dT are proliferating the d/min/mg tissue and d/min/kidney time-course curves are proportional to the total cell production following treatment.

*Intestine*—Intestinal-response deficit (IRD) was estimated by the integrated cell production (area under curve; AUC) after the second treatment as a percentage of AUC after a single treatment.

$$\mathrm{IRD} = \left[1 - \left(\frac{\text{AUC for multiple treatment}}{\text{AUC for single treatment}}\right) \times 100\%\right]$$

*Kidney.*—The renal -response deficit (RRD) was estimated by the integrated cell production after 100 mg/kg FA given at various times after DDP treatment as a percentage of that after 100 mg/kg FA in untreated kidneys. The ratios for cell production measured by both ct/min/kidney and LI were compared.

$$\mathrm{RRD} = \left[1 - \left(\frac{\text{AUC for multiple treatment}}{\text{AUC for single treatment}}\right) \times 100\%\right]$$

RESULTS

*Single treatment with* 8 *mg/kg DDP*

After a single injection of DDP, transient interruption in jejunal proliferation

Fig. 1.—Effect of a single dose of DDP (8 mg/kg) on the proliferative activity of the intestine and kidney in BDF1 mice. Each point represents mean (±s.e.) of the data from 5 animals.

was noted up to 36 h with subsequent compensatory hyperplasia between 48 and 144 h (Fig. 1). Cell production then returned to control levels (7 days) and remained so throughout the next 38 days of observation.

Unlike the intestine, the kidney, normally associated with low rates of cell production, was not immediately affected by a single injection of DDP. Within 48 h of treatment, however [$^3$H]dT incorporation into the kidney gradually increased, reaching a maximum (740% of untreated control) on Day 7 (Fig. 1). Proliferation gradually returned to nearly control levels by Day 45 (145% of control).

*Sequential treatment with* 8 *mg/kg DDP*

After the initial DDP treatment, a second dose of 8 mg/kg DDP was given to animals on Days 7, 14, 21 or 45. Intestinal proliferation at the time of the second treatment had returned to control levels. Recovery after the second DDP treatment was subnormal at all intervals up to 45 days (Fig. 2). Proliferative peaks after the second treatment occurred earlier (72 h) and were lower. Cell production over the 120 h period after treatment on Days 0+7 and Days 0+45 was about 65% of that after a single treatment, whilst for Days 0+14 and Days 0+21, cell production was 80% of groups receiving a single treatment (Table I).

Unlike the intestine, the proliferative activity in the kidney at the time of the second treatment was higher than control, untreated levels (Fig. 3). A second treatment on Day 7, when the kidney is at its proliferative maximum, produced a 60% reduction in [$^3$H]dT uptake by 24 h. During the next 96 h, [$^3$H]dT incorporation remained depressed, and in a subsequent experiment, treatment on Days

FIG. 2.—The effect of the interval between treatments during sequential DDP treatment (8 mg/kg/dose) on the proliferative activity of the intestinal epithelium. Zero time for each curve is the time of the last DDP treatment. Each point represents the mean (±s.e.) for 5 animals.

FIG. 3.—The effect of the interval between treatments during sequential DDP treatment on the [$^3$H]dT uptake in the kidney. Each point represents the percentage of the [$^3$H]dT incorporation value at the time of the final treatment ($t_0$). The absolute $t_0$ values (d/min/kidney ±s.e.) for each curve are given on the right of the figure and demonstrate the changing proliferative activity with time from a single DDP injection. Each point represents the data for 5 animals.

TABLE I.—*Response of the intestinal epithelium to multiple treatments with DDP*

| | Integrated cell production | |
|---|---|---|
| 2nd Treatment* (days) | d/min/mg† (%) | IRD‡ |
| 0 (control)§ | 100 | 0 |
| +7 | 67 | 33 |
| +14 | 82 | 18 |
| +21 | 82 | 18 |
| +45 | 69 | 31 |

* 8 mg/kg DDP.

† Area under the [$^3$H]dT d/min/mg wet wt curve.

‡ Response deficit calculated from the intestinal d/min/mg curve.

§ All animals received 8 mg/kg DDP at Day 0.

0 + 7 produced a 40% mortality of animals by Day 10 after treatment. A second treatment on Days 14 or 21 also depressed proliferation (by ≃40%) but, unlike the 0+7 group, recovery and compensatory hyperproliferation were seen between 96 and 120 h. A second treatment on Day 45 did not substantially reduce [$^3$H]dT incorporation. On the contrary there was a gradual *increase* in proliferative activity through 96 h, thereafter falling rapidly to [$^3$H]dT incorporation rates of 45% of the initial level by 120 h.

*Proliferative reserve in renal tissue during sequential treatment*

To measure the proliferative recovery potential in renal tissues after a single treatment with DDP, FA-induced tubular necrosis was used as an acute proliferative stimulus. After FA (100 mg/kg) treatments, the kidneys were yellow in colour with areas of focal haemorrhage. Histologically, the tubular epithelium was dilated and contained casts (areas not staining with H. & E.). Occasional necrotic and degenerative changes were seen in the tubular epithelial cells within 24 h of FA

Fig. 4.—[$^3$H]dT uptake in the kidney after 100 mg/kg FA as a function of time after DDP treatment. (●), untreated animals; (○), animals treated with 8 mg/kg DDP on Day 0. Each point represents the mean ±s.e. for 5 animals.

Fig. 5.—Renal tubular epithelial cell proliferation (LI) following 100 mg/kg FA as a function of time after DDP treatment. (●), untreated animals; (○), animals treated with 8 mg/kg DDP on Day 0. Each point represents the mean ±s.e. for 5 animals.

treatment. The [$^3$H]dT uptake in control kidneys was increased by 24 h after FA stress, with maximal incorporation at 72 h (Fig. 4). For all intervals, the proliferative renal response to FA stress was subnormal. The data suggested a recovery of the proliferative reserve between 7 and 14 days. However, a progressive decrease in compensatory proliferative response to FA was seen between 14 and 45 days.

Fig. 5 demonstrates the changes in the [$^3$H]dT labelling index (LI) for tubular epithelium in the renal cortex after FA stress (100 mg/kg). In the control animals, LI was significantly increased by 24 h, reaching a maximum (increased 120-fold) at 72 h and falling rapidly at 96 h. In

TABLE II.—*Response of the renal tubular epithelium to multiple treatments with DDP*

(Effect of FA proliferative stimulus after initial DDP treatment)

| Treatment* (days) | Integrated cell production (%) LI† | d/min/kidney‡ | RRD§ |
|---|---|---|---|
| 0 (control) | 100 | 100 | 0 |
| +7 | 59 | 48 | 46 |
| +14 | 87 | 87 | 13 |
| +21 | 59 | 34 | 53 |
| +45 | 29 | 24 | 74 |

* Time of FA stress following DDP treatment.
† Area under the [$^3$H[dT LI curve.
‡ Area under the [$^3$H]dT d/min/kidney curve.
§ Combined response deficit of LI and d/min/kidney response pretreated mice, LIs before FA stress were significantly higher than control. On Days 7 and 14 after DDP, the LI was 20 and 10 times the control, respectively. Not unlike the [$^3$H]dT uptake data (Fig. 4), the renal response to FA stress demonstrated a subnormal proliferative reserve for all intervals studied up to day 45 after DDP treatment.

If all tubular cells that took up [$^3$H]dT were subsequently to divide, the area under the d/min/kidney and LI time-course curves would be proportional to the total cell production during the experiments. Table II illustrates the changes in net cell production induced by FA as a function of the time after an initial DDP treatment. In general, the magnitude of cell production estimated from the LI curve was greater than that estimated by d/min/kidney. However, the relative deficits in integrated cell production calculated from these two indices were similar. The response deficits on Days 7 and 21 were similar. However, on Day 14 the response deficit was at a minimum, separating acute DDP renal effects from a significant and progressively increasing long-term reduction in renal proliferative reserve.

### DISCUSSION

The results from these studies demonstrate the response of two different normal tissues to cis-platinum II. In the rapidly proliferating intestinal epithelium, DDP produces a transient inhibition of cell production, followed by a compensatory recovery and a return to steady-state control levels 7 days after treatment (Fig. 1). The time course and magnitude of this response have been found to be dose-dependent (Phillips & Fu, 1976; Burholt et a ., 1979; Luk *et al.*, 1979), suggesting that the drug alone is cytotoxic to the intestinal crypt cells. In addition, DDP has been found to enhance the gastro-intestinal radiosensitivity in combined radiation + drug studies (Luk *et al.*, 1979; Schenken *et al.*, 1979*a*).

By comparison, renal-tubule cell proliferation is not markedly affected by DDP treatment (Fig. 1). Incorporation of [$^3$H]dT is only slightly depressed during the first 24 h after treatment, increasing after 3 days and reaching a 6-fold increase on Day 7. Taylor *et al.* (1976) have reported similar observations in rats treated with 4·0 mg/kg DDP. However, they observed an initial marked inhibition of DNA synthesis before a 16-fold increase by Day 7. Cell proliferation in the kidney under non-stress conditions as monitored by d/min/kidney reflects proliferation of transitional and squamous epithelium rather than tubular epithelium, which is extremely low. This may explain the discrepancy between the initial renal response to DDP reported here and that observed by Taylor *et al.* (1976). In addition, there may be interspecific differences in drug dose as suggested by Litterst *et al.* (1979).

Treatments after the first dose of DDP led to suboptimal recovery of the jejunal epithelium for intertreatment times up to 45 days (Fig. 2). Both the magnitude and time of compensatory proliferative peaks were reduced. While steady-state proliferative conditions were restored by Day 7, the response deficits calculated for subsequest treatments up to 45 days (Table I) suggested that DDP in sequential protocols would enhance the intestinal drug sensitivity. Second treatments on Days 7 or 45 were less well tolerated than those on Days 14 or 21. Similar enhanced drug sensitivity was found in the kidney after an initial treatment with DDP (Figs 4 & 5; Table II). Again a subsequent treatment on Day 14 appeared more tolerable than after the other treatment intervals. In the kidney, the distinction between early (acute) and delayed enhancement of sensitivity was more apparent.

Delayed complications after both drug and radiation therapy have been recognized in a number of normal tissues (Phillips & Fu, 1976; Nygaard *et al.*, 1976). Schenken *et al.* (1979*b*), have recently demonstrated enhanced gastrointestinal radiosensitivity by Adr, where the drug and radiation treatments were separated by 14–49 days. Similarly to our observations for sequential drug treatment, they observed that radiosensitivity diminished from the time of drug treatment until 14 days, and then progressively increased in severity for up to 49 days. Enhanced tissue sensitivity, when intertreatment times are short is probably a direct result of the interaction of the treatment modalities on the cell-proliferation kinetics of the tissue. Enhanced sensitivity arising from longer treatment intervals, however, is most probably a manifestation of reduced proliferative reserves in the normal cell-renewal systems (Schenken *et al.*, 1979*b*; Braunschweiger *et al.*, 1980; Morley, 1980).

Dentino *et al.* (1978) have reported permanent, nonspecific functional renal injury in patients treated with DDP. They suggested that "... while renal injury remained subclinical, future courses of DDP could lead to clinically important renal failure". From the results presented here, increasing the intertreatment time from 7 to 14 days appeared to permit proliferative recovery of the renal tissue (Fig. 3). None the less, under similar time considerations, a single dose of 8 mg/kg DDP produced greater restriction on the capacity of the proliferative reserve of the kidney to mount a compensatory response to FA-induced renal tubular necrosis (Figs 4 & 5; Table II). Under continued treatment with DDP, as suggested by Dentino, subclinical changes in the renal proliferative reserve could be compounded, leading to chronic renal failure.

The question remains: "What is the basis for drug-induced limitation of proliferative reserve in normal tissues?" Earlier (Schenken *et al.*, 1979*b*; Kovacs *et al.*, 1981) it was suggested that the effect was a manifestation of latent damage to the stem-cell compartment brought about by sequestration of sublethal levels of drug. Alternatively, damage could be accumulated in a secondary support system such as the tissue vasculature. We have observed that on Day 14 the capacity of DDP-treated kidneys to mount a compensatory proliferative response to FA damage is greater than on Days 7, 21 or 45. The half-life of DDP retention in kidney tissue has been reported as 8·4 days (Taylor *et al.*, 1976) with significant levels retained tightly bound to tissue protein and nucleic-acid bases (Taylor *et al.*, 1973). This could explain the apparent recovery of early damage (Days 0–14) shown in Fig. 5, where near-normal levels of cell production (87%; Table II) occur after FA stress. However, if drug retention alone were responsible for the reductions in proliferative reserve, and near-normal responses occurred at Day 14 after drug treatment, similar or even improved responses to FA would be expected on Days 21 and 45. Rather it appears that a secondary effect, not related to the immediate drug inhibition of the proliferative reserve, develops at these later times, and has been found up to 120 days after the initial treatment (Braunschweiger *et al.*, 1980, and unpublished).

One could argue that when the kidney undergoes proliferative recovery, the cell population of the proliferative reserve is reduced, thus reducing the overall ability of the tissue to respond to further proliferative stress. Experimentally induced renal hypertrophy has, however, been extensively studied in both mice and rats (Baserga *et al.*, 1968; Byrnes *et al.*, 1972*a*,*b*) and these data suggest that the kidney is capable of responding to a second stress stimulus (Threlfall *et al.*, 1967). Alternatively, long-term exposure to even low levels of drug could permanently damage the renal microenvironment, which in turn could have a net antiproliferative effect. Dentino *et al.* (1978) have observed a regular and persistent decrease in the glomerular filtration rate of patients after the second course of DDP; they noted segmental cellular necrosis in the proximal and distal tubules at 3–6 weeks after treatment, and after 5 months focal interstitial fibrosis was evident as well as tubular atrophy and dilation.

It is as yet unclear whether the underlying mechanisms of the long-term toxicities from DDP treatment are similar for intestinal and renal-tubular epithelium. However, from a clinical standpoint, the answer to such a question takes on new importance, especially for continuing or recurring therapy. Studies are continuing to elucidate the nature of, and amelioration of, these potentially harmful effects.

### REFERENCES

BASERGA, R., THATCHER, D. & MARZI, D. (1968) Cell proliferation in mouse kidney after a single injection of folic acid. *Lab. Invest.*, **19**, 92.

BRAUNSCHWEIGER, P. G., KOVACS, C. J., SCHENKEN, L. L., SCHIFFER, L. M. & PUGH, R. P. (1980) Residual hematopoietic and renal injury in mice after DDP. *Proc. Am. Soc. Clin. Oncol.*, **21**, 334.

BRUNO, S., POSTER, D. S., HIGBY, D. J., BURKE, P. & NEITTLEMAN, S. (1980) Parameters of nephrotoxicity in relation to the administration of cis-DDP. *Proc. Am. Assoc. Cancer Res.*, **21**, 150.

BURHOLT, D. R., HAGEMANN, R. F., SCHENKEN, L. L. & LESHER, S. (1977) Influence of adriamycin and adriamycin–radiation combination on jejunal proliferation in the mouse. *Cancer Res.*, **21**, 22.

BURHOLT, D. R., SCHENKEN, L. L., KOVACS, C. J. & HAGEMANN, R. F. (1979) Response of the murine gastrointestinal epithelium to cis-dichlorodiammine platinum. II: Radiation combinations. *Int. J. Radiat. Oncol. Biol. Phys.*, **5**, 1377.

BYRNES, K. A., GHIDONI, J. J. & MAYFIELD, E. D. (1972*a*) Response of the rat kidney to folic acid administration. I. Biochemical studies. *Lab. Invest.*, **26**, 184.

BYRNES, K. A., GHIDONI, J. J., SUZUKI, M., THOMAS, H. & MAYFIELD, E. D. (1972*b*) Response of the rat kidney to folic acid administration. II. Morpholic studies. *Lab. Invest.*, **26**, 191.

DENTINO, M., LUFT, F. C., YUM, M. N., WILLIAMS, S. D. & EINHORN, L. H. (1978) Long term effect of cis-diamminedichloride platinum (C-DDP) on renal function and structure in man. *Cancer*, **41**, 1274.

DOUPLE, E. B., RICHMOND, R. C. & LOGAN, M. E. (1977) Therapeutic potentiation in a mouse mammary tumor and an intracerebral rat brain tumor by combined treatment with cis-dichlorodiamine platinum (II) and radiation. *J. Clin. Hemato. Oncol.*, **7**, 585.

FREEMAN, A. I., ETTINGER, L. J. & BRECHER, M. C. (1979) Cis-dichlorodiammine platinum (II) in childhood cancer. *Cancer Treat. Rep.*, **63**, 1615.

GONZALES-VITALE, J. C., HAYES, D. M., CVITKOVIC, E. & STERNBERG, S. S. (1977) The renal pathology in clinical trials of cis-platinum (II) diammine dichloride. *Cancer*, **39**, 1362.

HIGBY, D. J., WALLACE, H. J. & ALBERT, D. J. (1974) Diaminodichloro-platinum: A phase I study showing responses in testicular and other tumors. *Cancer*, **33**, 1219.

KOVACS, C. J., SCHENKEN, L. L., EVANS, M. E. & BURHOLT, D. R. (1981) Enhanced adriamycin-induced gastrointestinal radio-sensitivity in tumor-bearing animals. *Int. J. Radiat. Oncol. Biol. Phys.*, **7**, 1389.

LEHANE, D., WINSTON, A., GRAY, R. & DASKEL, Y. (1979) The effect of diuretic pre-treatment on clinical, morphological, and ultrastructural cis-platinum induced nephrotoxicity. *Int. J. Radiat. Oncol. Biol. Phys.*, **5**, 1393.

LITTERST, C. L., LEROY, A. F. & GUARINE, A. M. (1979) Disposition and distribution of platinum following parenteral administration of cis-dichlorodiammine platinum (II) to animals. *Cancer Treat. Rep.*, **63**, 1485.

LUK, K. H., ROSS, G. Y., PHILLIPS, T. L. & GOLDSTEIN, L. S. (1979) The interaction of radiation and cis-diamminedichloroplatinum (II) in intestinal crypt cells. *Int. J. Radiat. Oncol. Biol. Phys.*, **5**, 1417.

MORLEY, A. (1980) Residual marrow damage from cytotoxic drugs. *Aust. N.Z. J. Med.*, **10**, 569.

NYGAARD, K., SMITH-ERICHSEN, N., HATLEVOLL, R. & REFSUM, S. B. (1976) Pulmonary complications after bleomycin, irradiation and surgery for esophageal cancer. *Cancer*, **37**, 1186.

PHILLIPS, T. L. & FU, K. K. (1976) Quantification of combined radiation therapy and chemotherapy effects on critical normal tissue. *Cancer*, **37**, 1186.

PRESNOV, M. A., KONOVALOVA, A. L., ROMANOVA, L. F., SOFINA, Z. P. & STETSENKO, A. I. (1978) Chemotherapy of transplanted mouse tumors with cis-dichlorodiammineplatinum (II) alone and in combination with sarcolysin. *Cancer Treat. Rep.*, **62**, 705.

SCHENKEN, K. L., BURHOLT, D. R., HAGEMANN, R. F. & KOVACS, C. J. (1979*a*) Combined modality oncotherapies: Cell kinetic approaches for avoidance of gastrointestinal toxicity. In *Frontiers of Radiation Therapy and Oncology* (Ed. Veath). Baltimore: University Press. Vol. 13, p. 82.

SCHENKEN, L. L., BURHOLT, D. R. & KOVACS, C. J. (1979*b*) Adriamycin–radiation combinations: Drug-induced delayed gastrointestinal radiosensitivity. *Int. J. Radiat. Oncol. Biol. Phys.*, **5**, 1265.

TAYLOR, D. M., JONES, J. D. & ROBINS, A. B. (1973) Metabolism of platinum $^{14}C$-ethylendiamine dichloride in the rat. *Biochem. Pharmacol.*, **22**, 833.

TAYLOR, D. M., TEW, K. D. & JONES, J. D. (1976) Effects of cis-dichlorodiammine platinum (II)

20

on DNA synthesis in kidney and other tissues or normal and tumor bearing rats. *Eur. J. Cancer*, **12**, 249.

THRELFALL, G., TAYLOR, D. M. & BUCK, A. T. (1967) Studies of the changes in the growth and DNA synthesis in the rat kidney during experimentally induced renal hypertrophy. *Am. J. Pathol.*, **50**, 1.

WALLACE, H. J. & HIGBY, D. J. (1974) Phase I evaluation of cis-platinum (II) diammine dichloride (PPD) and a combination of PDD plus adriamycin. In *Platinum Coordination Complexes in Cancer Chemotherapy* (Eds Conners & Roberts). New York: Springer-Verlag. p. 145.

WELSCH, C. W. (1971) Growth inhibition of rat mammary carcinoma induced by cis-platinum diamminodichloride. II. *J. Natl Cancer Inst.*, **47**, 1070.

YOGODA, A., WATSON, R. C., KEMENY, N., BORZELL, W., GRABSTALD, H. & WHITMORE, W. F. (1978) Diamminedichloride platinum II and cyclophosphamide in treatment of advanced urothelial cancer. *Cancer*, **41**, 2121.